# BAX, VDAC, Gasdermin, and Permeability Transition Pores in Inflammatory Mitochondrial DNA Release: Is This the “Hole” Story?

**DOI:** 10.3390/cells15201839

**Published:** 2026-10-10

**Authors:** Naibo Zhang, Daniel D. Freeman, Brian M. Polster

**Affiliations:** Department of Anesthesiology, Center for Shock, Trauma and Anesthesiology Research, and Program in Neuroscience, Baltimore, MD 21201, USA; naibo.zhang@som.umaryland.edu (N.Z.); daniel.freeman@som.umaryland.edu (D.D.F.)

**Keywords:** mtDNA, mPTP, mitophagy, cGAS, STING, NLRP3, inflammasome, MDV, MLKL, apoptosis

## Abstract

Disease-modifying neuroprotective therapies for traumatic brain injury (TBI), stroke, and age-related neurodegenerative disorders have proven elusive. Interest in mitochondrial outer membrane permeabilization mechanisms as a drug target evolved from the 1990s discovery of BAX/BAK-mediated mitochondrial cytochrome *c* release as an apoptotic cell death trigger. However, enthusiasm waned as subsequent findings revealed that apoptosis is not the dominant cell death mechanism in the mature brain. The more recent discovery of mitochondrial DNA (mtDNA) as an inflammatory trigger produced renewed interest in mitochondrial permeability pathways. In particular, the DNA-sensing molecule cGAS and its downstream effector STING were implicated in the pathogenesis of several neurodegenerative conditions, including Alzheimer’s disease (AD), Parkinson’s disease (PD), amyotrophic lateral sclerosis (ALS), and TBI. Nevertheless, these findings raised a puzzling question: how does mtDNA, which resides in the mitochondrial matrix surrounded by double membranes, escape to initiate inflammatory responses without triggering cytochrome *c*-dependent cell death? Here, we review the many proposed mechanisms for mtDNA release, including several that do not require mitochondrial membrane holes, and discuss advances toward pharmacological targeting of these pathways. Identification of the specific mtDNA efflux pathway(s) proximal to maladaptive neuroinflammatory responses may yield new treatment strategies for AD, TBI, and other neurological diseases.

## 1. Introduction

The mitochondrion is a double-membraned organelle that produces adenosine triphosphate (ATP) in response to cellular energy needs [1]. Mitochondria contain their own genetic material, a 16,569 base pair (bp) genome, making them unique among mammalian organelles [2]. Unlike nuclear DNA, which is compactly arranged into chromosomes, mitochondrial DNA (mtDNA) is circular, present in multiple copies, and packed into protein–DNA complexes called nucleoids [3]. Mitochondrial DNA sequences were identified in nuclear genomes several decades ago, supporting the theory that genes were transferred from ancestral prokaryotic endosymbionts (the precursors of mitochondria) into the nuclei of eukaryotic cells during evolution [4,5,6]. In 1990, Thorsness and Fox provided one of the first experimental demonstrations in living cells that mtDNA can escape from mitochondria, showing mtDNA relocalization to the nucleus in yeast [7]. Twenty years later, it was discovered that mtDNA is released into circulation in people following tissue trauma [8]. MtDNA release to the cytosol, either as nucleoids in which transcription factor A, mitochondrial (TFAM) packages and organizes circular mtDNA or as short linear DNA fragments, has now been observed in conjunction with increased mitochondrial membrane permeability in many human diseases [9,10]. Emerging evidence indicates that mtDNA translocation, the movement of mtDNA from mitochondria to other cellular compartments, is not simply an unregulated consequence of mitochondrial compromise, but plays an active role in intracellular, or even cell-to-cell, signaling. Activation of multiple inflammatory cascades has been attributed to non-conventional (i.e., transcription-/translation-independent) mtDNA signaling [8,11,12,13,14,15]. This signaling is implicated in disease resolution or progression, depending on the illness and the cell type.

Radiation [16,17,18,19], bacterial or viral infection [11,12], and mechanical damage to tissues [8] are established causes of mtDNA release to the cytosol and/or extracellular space. In the case of radiation, mtDNA release may be initiated by direct damage; fragments of mtDNA were observed in the cytosol of fractionated brain as early as one hour after irradiation of mice [16]. However, the participation of upstream signaling factors responding to radiation has not been excluded. In the case of infection, pathogen-derived molecules engage members of the toll-like receptor (TLR) family, as well as additional plasma membrane or intracellular receptors, to trigger signal transduction cascades leading to altered mitochondrial permeability and function. However, many of the same receptors that detect pathogen-associated molecular patterns (PAMPs) also show affinity to a diverse group of endogenous molecules called damage-associated molecular patterns (DAMPs). These endogenous molecules are sequestered away from the receptors in healthy cells, serving a variety of non-immune functions, but propagate inflammation upon release [20,21]. Pyroptosis is a mediator of DAMP release in infectious disease [22]. It is a lytic form of programmed cell death that depends on gasdermin pore-forming proteins to perforate the plasma membrane [23,24,25,26] and ninjurin-1 (NINJ1) to induce membrane rupture [27]. However, DAMP release also occurs via pyroptosis-independent mechanical membrane damage, such as during necrotic cell death caused by brain trauma or stroke [28,29], and this death may also be NINJ1-regulated [30,31].

Inflammatory pathway activation triggered by DAMPs in non-infectious disease states is referred to as “sterile inflammation” [20,21]. There is compelling evidence that sterile inflammation contributes to neurodegeneration following traumatic brain injury (TBI) [32,33,34,35]. High mobility group box protein 1 (HMGB1) is one DAMP that is elevated in human cerebrospinal fluid for days after TBI and it appears to contribute to both the acute and subacute phases of TBI-induced cognitive dysfunction [36,37]. Though extensive evidence suggests that maladaptive inflammatory pathway activation also contributes to the pathogenesis of many chronic neurodegenerative disorders, including Alzheimer’s disease (AD), Parkinson’s disease (PD), frontotemporal dementia (FTD), and amyotrophic lateral sclerosis (ALS) [38], the proximal causes of neuroinflammation in these diseases are less clear. It is possible that intracellular mtDNA redistribution is one of the initiating events, as mitochondrial disruption due to misfolded or mislocalized proteins like TAR DNA-binding protein 43 (TDP-43), α-synuclein, and amyloid β peptides is reported [39,40,41,42]. In addition to acting as an intracellular inflammatory mediator downstream of triggers like HMGB1 or misfolded proteins, mtDNA may also function as a conventional DAMP by activating cell-surface receptors when released into the extracellular space [8].

## 2. Parallels to Cell Death Signaling

Prior to the discovery of mtDNA’s role in inflammatory signaling, mitochondrial membrane permeabilization was thought to contribute to neurodegeneration primarily by causing mitochondrial bioenergetic dysfunction and cell death [43,44]. Elegant studies in the mid-to-late 1990s illuminated an unexpected role for the electron transport chain component cytochrome *c* in apoptosis initiation. Upon mitochondrial escape, cytochrome *c* triggers this intrinsic programmed cell death pathway by assembling the apoptosome protein complex in the cytosol, resulting in caspase-9 protease activation [45,46]. This initiator caspase then activates effector caspases, such as caspase-3 and caspase-7, which orchestrate the dismantling of the cell. Knock-in of an apoptosome-assembly deficient but electron transfer-competent cytochrome *c* mutant in mice established cytochrome *c*’s role in apoptosis in vivo [47].

Work over the next two decades began to indicate an important role for mitochondria in inflammatory signaling. In the hunt for the mitochondrial signal(s) mediating inflammatory activation, initial studies revealed the participation of mitochondrial reactive oxygen species (ROS) production [48,49]. Later, it was found that mtDNA, when released into the cytosol, may help to nucleate an “inflammasome” complex consisting of NLR family pyrin domain containing 3 (NLRP3), NIMA-related kinase 7 (NEK7), the adaptor protein apoptosis-associated speck-like protein containing a CARD (ASC), and pro-caspase-1 [12,50], analogous to cytochrome *c*-mediated apoptosome formation that also contains an adaptor (apoptotic peptidase activating factor 1 (APAF-1)) and a pro-caspase. Potentially explaining mitochondrial ROS participation, oxidized mtDNA was proposed to be the specific trigger [15], which may bind to the NLRP3 pyrin domain [51] or intrinsically disordered region [52]. Additional evidence indicated that newly synthesized mtDNA is required for NLRP3 inflammasome activation [14], which was hypothesized to be more susceptible to oxidation prior to nucleosome incorporation. Nevertheless, the involvement of mtDNA in NLRP3 inflammasome activation remains incompletely understood, as not all data points to a requirement for mtDNA [53], or even for mitochondrial ROS [54].

Evidence that the well-established DNA-sensing molecule cyclic GMP-AMP synthase (cGAS) and its effector stimulator of interferon genes (STING) are required for NLRP3 activation, at least in some cells, may help to explain the role of mtDNA in inflammasome activation [55]. Other evidence points to a role for the orphan intracellular receptor neuron-derived clone 77 (NUR77, also called nuclear receptor subfamily 4 group A member 1 (NR4A1)), which binds both lipopolysaccharide (LPS) and mtDNA to promote non-canonical NLRP3 inflammasome activation downstream of the activation of caspase-11, the mouse homologue of human caspase-4 [56]. It is possible that interacting signaling pathways, check points, and feedback loops contribute to the divergent conclusions regarding the roles of mtDNA, mitochondrial ROS, and cGAS-STING signaling in inflammasome activation.

Importantly, mtDNA release is observed not only during apoptotic cell death when large pores in the mitochondrial outer membrane (MOM) allow cytochrome *c* and other mitochondrial proteins to escape, but also in cases of moderate mitochondrial stress. In these cases, mtDNA release can trigger immune responses without activating caspases to a level that results in cell death [57]. Stimuli that activate NLRP3 remodel mitochondrial cristae architecture, appearing to trap cytochrome *c* within cristae compartments. Consequently, mtDNA release may occur in some settings without efficient mobilization of cytochrome *c* from mitochondria, allowing inflammatory signaling to prevail over apoptosis [58].

Knowledge of the molecular mtDNA release mechanisms and the factors dictating which inflammatory effector pathway is recruited (either with or without ultimate cell death) has advanced substantially within the last few years but remains incomplete. Initial research focused primarily on the same molecular pathways implicated in cytochrome *c* release during apoptosis or necrosis, previously described as cell death “gateways” [59]. An updated account of these membrane pores in the context of mtDNA release and inflammation is given below, followed by a discussion of more recently implicated pathways, including roles for gasdermin family proteins, endosomes, and mitochondria-derived vesicles.

## 3. BAX/BAK-Mediated Mitochondrial Outer Membrane (MOM) Macropores

BCL-2-associated X protein (BAX) and BCL-2 homologous antagonist/killer (BAK) are multi-domain B-cell lymphoma (BCL-2) family proteins of partly overlapping function required for the cytochrome *c*/caspase-dependent intrinsic apoptotic pathway in mammalian cells [60]. BAK is a resident mitochondrial outer membrane protein bound to voltage-dependent anion channel (VDAC) 2 [61], while BAX is primarily cytosolic until its mitochondrial translocation and outer membrane integration are triggered by an activating stressor [62]. Conformational changes promoted by BCL-2 homology 3 (BH3) domain-only “activator” proteins such as BH3 interacting domain death agonist (BID) or BCL-2 interacting mediator of cell death (BIM) cause BAX and BAK to oligomerize and form proteolipidic pores in the mitochondrial outer membrane, creating a conduit to the cytosol for cytochrome *c* and other soluble intermembrane space proteins (Figure 1) [63,64,65,66,67,68,69]. Several studies indicate that this occurs independently of increased inner membrane permeability, e.g., mitochondrial permeability transition pore (mPTP) opening, when the pores are initially formed [63,65,67,68,69,70].

Anti-apoptotic members of the protein family, including founding member BCL-2, as well as B cell lymphoma X long (BCL-X_L_), B cell lymphoma W (BCL-W), and myeloid cell leukemia 1 (MCL-1), inhibit mitochondrial outer membrane permeabilization (MOMP) by multiple mechanisms. These include sequestering the BH3-only activators [64,71,72], antagonistically binding to BAX/BAK themselves at the mitochondria [71,73], and, for BCL-X_L_, retro-translocating BAX from the mitochondria to the cytosol [74,75]. Central nervous system (CNS) expression of BAX and BAK decreases during brain maturation as the period of developmental apoptosis comes to an end [76,77]. BAX appears to be the major pro-apoptotic player in neurons [43,76,78] but less is known on BAX vs. BAK dominance in other brain cell types such as microglia, the innate immune cells of the CNS.

While BAX/BAK macropores have been studied extensively since the late 1990s in the context of cytochrome *c* release, a pair of studies published in 2018 demonstrated that the outer membrane pores can also participate in mtDNA release [79,80]. Advanced imaging methods unavailable when BAX/BAK pores were first discovered, including lattice light-sheet microscopy, correlated light and electron microscopy, and 3D structured illumination microscopy, were used to show that the mitochondrial inner membrane (MIM) herniates through BAX/BAK macropores in the MOM and sometimes loses integrity, resulting in mtDNA and nucleoid protein extrusion [79,80].

BAX/BAK macropores do not form in the MIM and, therefore, additional processes are required to permeabilize the extruding MIM. The mechanisms of herniated MIM permeabilization during apoptosis are unclear but may involve gasdermin family proteins, which will be discussed below. However, it was recently discovered that the antibacterial protein apolipoprotein L3 (APOL3) is the effector that mediates herniated MIM integrity loss downstream of lysosomal damage-triggered BAX/BAK macropore formation in interferon-gamma-primed immune cells [81]. In this specific context, only some mitochondria are permeabilized by BAX/BAK, resulting in a phenomenon that has been termed “incomplete” or “minority” MOMP [82,83]. The enzyme phospholipid scramblase-3 (PLSCR3) causes externalization of the MIM-specific lipid cardiolipin, which recruits APOL3 to the herniated membrane [81]. APOL3 then initiates mtDNA release through a detergent like-solubilization of cardiolipin, analogous to its mode of bacterial attack.

Minority MOMP is not a phenomenon exclusive to immune cells, as it is also observed under sub-lethal or low-level apoptotic conditions [82,83] and as a feature of cellular senescence [57]. Moderate cytochrome *c* release accompanied by mtDNA release and inflammatory cytokine secretion occurs during minority MOMP. Cells have multiple checkpoints to prevent cell death execution, including the inhibitor of apoptosis protein (IAP) family that can suppress caspase activation downstream of cytochrome *c* release [84,85]. Such checkpoints may favor inflammatory signaling over apoptosis in an incomplete MOMP scenario.

## 4. Mitochondrial Permeability Transition Pore (mPTP)

The mitochondrial permeability transition pore (mPTP) is a high-conductance channel in the mitochondrial inner membrane that opens in response to matrix Ca^2+^ overload [86,87,88] (Figure 2). The channel is desensitized to opening by the drug cyclosporin A (CsA) [89,90,91], which is one of its defining characteristics. ROS are a second channel trigger. Oxidative stress lowers the threshold for mPTP opening, at least in part through the oxidation and cross-linking of critical protein thiols [92,93,94,95]. Transient mPTP opening, or “flickering,” has been observed in various contexts and is thought to help limit toxic matrix calcium and ROS accumulation [96,97,98,99,100,101,102]. However, sustained mPTP opening leads to ion equilibration across the MIM, which induces osmotic swelling and MOM structural rupture [86,87,88]. The ensuing release of mitochondrial intermembrane space contents following this physical disruption is thought to be non-selective.

The molecular identity of the mPTP remains a mystery, but channel formation is thought to involve multiple proteins residing at contact sites between the mitochondrial inner and outer membranes [103]. Cyclophilin D (CypD), a cis–trans peptidyl prolyl isomerase that is the target of CsA [89,90,91,104], is a well-established regulator [105,106,107]. CsA suppresses permeability transition less effectively in brain mitochondria than in liver mitochondria [108,109], potentially reflecting heterogeneity in CypD expression across different brain cell types [110,111]. Early studies additionally implicated VDAC, the adenine nucleotide translocator (ANT), and the mitochondrial phosphate carrier (PiC) as mPTP channel components [94,112,113,114,115,116,117,118]. However, genetic ablation studies did not support a universal requirement for any of these candidates despite establishing ANT and PiC alongside CypD as mPTP opening “sensitizers,” meaning that knockout increases the calcium threshold for permeability transition induction [119,120,121,122,123].

The hypothesis that the mPTP is formed by an altered conformation of the F1Fo ATP synthase or one or more of its subunits has dominated the literature over the last decade [103]. Isolated ATP synthase reconstituted into artificial bilayers generates high-conductance currents in response to mitochondrial calcium overload [124,125]. Moreover, genetic manipulation of the F1Fo ATP synthase c-subunit [126] and stabilization of its dimers [127] were both found to reduce mPTP opening following mitochondrial calcium overload. Additional studies found that c-subunit-deficient human HAP1 cells have a low-conductance CsA-sensitive channel that causes mitochondrial depolarization but not swelling in response to calcium overload [128,129]. This led to the suggestion that the ANT may form a separate pore in the absence of the ATP synthase channel. However, evidence from another study supported a cooperative requirement for ANT and the ATP synthase in mPTP formation [130]. Despite these intriguing ATP synthase-mPTP links, genetic knockout studies from multiple labs have challenged the idea that the mPTP pore component resides in the ATP synthase [131,132,133,134,135], with one study showing mPTP persistence in human mitochondria devoid of an assembled ATP synthase [133] and another suggesting that the ATP synthase is instead a negative regulator of mPTP opening [134]. Within the last two years, independent studies provided evidence that the mitophagy regulator NLR family member X1 (NLRX1) is required for mPTP opening in specific screening/knockout systems, with mechanism and generalizability remaining to be defined [135,136,137].

While the mPTP is not required for BAX/BAK-induced cytochrome *c* release [63,65,68,69,70], there is evidence from multiple laboratories that its opening is responsible for mtDNA release in some contexts. A 2004 study provided evidence that small linear mtDNA fragments can escape through the pore [138]. An mPTP size of 2–3 nm has been estimated based on its electrophysiological conductance [90,139,140] and ability to pass globular small molecules up to 1.5 kilodaltons [88,141]. Because linear double-stranded DNA has a diameter of approximately 2 nm [142], linear mtDNA fragments could potentially traverse the pore despite possessing molecular masses that greatly exceed the 1.5 kilodalton solute exclusion limit. Consistent with this possibility, Patrushev et al. found that calcium addition to isolated mouse liver mitochondria caused mtDNA release alongside decreased membrane potential and swelling, all of which were reversed by CsA [138]. Moreover, shortly thereafter García et al. showed that CsA treatment prevented the appearance of mtDNA fragments in supernatant following the exposure of isolated rat liver mitochondria to calcium and oxidative stress produced by Fe^2+^ plus hydrogen peroxide [143]. CsA failed to inhibit mtDNA fragmentation, as seen by evaluating mitochondrially retained mtDNA on an agarose gel, supporting suppression of mtDNA efflux by mPTP inhibition rather than mtDNA protection from oxidative stress as CsA’s mechanism of action.

A later study by the same group provided evidence that mtDNA fragments released by the mPTP have a specific cutoff size potentially as low as ~700 bp [144]. This was done by sedimenting mitochondria following permeability transition and using various polymerase chain reaction (PCR) primers to amplify mtDNA regions of different lengths from the supernatant. Release of a DNA fragment containing the full-length *Mt-nd3* gene (348 bp) was detected whereas only 700 bp or 568 bp *Mt-co1* or *Mt-cyb* pieces, respectively, but not full-length *Mt-co1* (1544 bp) or *Mt-cyb* (1143 bp) genes, were seen in the supernatant. Furthermore, the *Mt-atp6*, *Mt-atp8*, *Mt-co3*, *Mt-co2*, and *Mt-co3* genes were present in a single large DNA fragment that failed to escape mitochondria, suggesting that the fragmentation of mtDNA by oxidative stress is selective, and that the mPTP size further controls what DNA escapes. Release of the intermembrane space enzyme adenylate kinase was observed but the release of the matrix enzyme malate dehydrogenase was not [144], consistent with outer membrane rupture but an intact inner membrane under the experimental conditions.

While these results suggest that mPTP opening is insufficient to release the large mtDNA nucleoids observed to escape in BAX/BAK-macropore-containing apoptotic cells, later studies demonstrated that calcium-induced mitochondrial permeabilization can progress to inner membrane fragmentation/rupture, both in isolated mitochondria [145] and in cells [146]. Gamma-irradiation-induced release of 1841 bp and 10,090 bp mtDNA fragments was observed in mouse brain homogenate ex vivo, with only the 1841 bp fragment detectable after one hour of 5 Gy mouse irradiation and both fragments appearing after five hours [16]. Based on the pore-size estimates reported by García et al. [144], neither of these mtDNA fragments should permeate the mPTP, suggesting that mPTP can progress to more extensive MIM permeabilization in vivo or that additional membrane-disruptive mechanisms contribute to radiation-induced mtDNA release. Importantly, because most mechanistic studies of mtDNA release have been performed in non-neural cells or isolated mitochondria derived from non-CNS tissues, brain-specific studies are needed to determine whether the pathways governing mtDNA release in the CNS differ from those identified in peripheral tissues, particularly given the well-established differences in mPTP regulation and sensitivity between brain and liver mitochondria [108,109].

Finally, it is notable that when using the endoplasmic reticulum (ER) calcium-releasing compound 2,4,6-trimethyl-N-(m-3-trifluoromethylphenyl) benzenesulfonamide (m-3M3FBS) to cause mitochondrial inner membrane permeabilization/fragmentation in cells, Quarato et al. found that CsA was protective by inhibiting ER calcium release upstream of MIM permeabilization and CypD knockout did not show the same protection [146]. Using a photoactivatable CsA analogue possessing a biotin moiety, Quarato et al. affinity-purified not only CypD but 86 additional proteins, consistent with the possibility that CsA has many cellular targets. These results caution against using CsA sensitivity as the sole criterion to implicate mPTP involvement in mtDNA release in cells. An early study of macrophage NLRP3 inflammasome activation further illustrates this point; NLRP3 inflammasome activation was inhibited by CsA, whereas CypD knockout macrophages showed normal NLRP3 activation in response to multiple activating stimuli [147].

## 5. VDAC Oligomer-Mediated MOM Pores

Although MOM rupture following sustained mPTP opening provides an obvious pathway for mtDNA fragments to reach the cytosol after they pass through the pore, there is evidence that mtDNA can escape into the cytosol even in cases where mPTP opening is transient, e.g., in senescent cells [148]. MtDNA release mediated by transient mPTP opening would presumably require an outer membrane conduit. As a highly abundant voltage-dependent anion channel that normally permeates ions and small molecules across the MOM, VDAC was a natural candidate for participating in mtDNA efflux.

Prior evidence using chemical crosslinkers suggested that VDAC oligomerizes during apoptosis to form a large MOM pore distinct from BAX/BAK macropores [149]. However, genetic ablation studies indicate that VDACs are not required for calcium-, oxidative-stress-, or BAX-dependent cell death [119], raising the possibility that VDAC oligomers serve another function. In an endonuclease G knockout cellular model exhibiting elevated cytosolic mtDNA and constitutive immune signaling, Kim et al. found oligomerized VDAC pores formed in response to mitochondrial ROS despite maintained cell viability [150]. The detected cytosolic mtDNA was enriched for small-size D-loop mtDNA fragments, consistent with García et al.’s sizing studies of mPTP-mediated mtDNA extrusion [144]. Accordingly, the cytosolic mtDNA level was decreased by either the mPTP inhibitor CsA or the putative VDAC inhibitor VBIT-4 [151], suggesting that the mtDNA fragments transit through both inner and outer membrane gates rather than escape through ruptured membranes [150] (Figure 3). These findings suggested a role for VDAC oligomers downstream of transient mPTP opening, distinct from cell death signaling functions.

Challenging the VDAC-oligomerization-mtDNA release model, emerging evidence indicates that VBIT-4 may not be a specific inhibitor of VDAC oligomerization, as it appears to have VDAC-independent effects on mitochondrial membrane structure [152]. Nonetheless, Kim et al. also showed that hydrogen-peroxide-triggered mtDNA release in wild-type cells is attenuated by knocking out *Vdac1*, *Vdac3*, or the two genes together [150], providing complementary genetic support for the results with the putative VDAC oligomerization inhibitor.

Kim et al. additionally proposed that mtDNA fragments serve as a scaffold to promote VDAC oligomerization, identifying three positively charged amino acids within VDAC’s N-terminus important for mtDNA binding and oligomerization. A later study found that mtDNA is not required for VDAC oligomerization triggered by inflammasome-activating stimuli in mouse macrophages but reproduced findings of a CsA- and VBIT-4 sensitive mtDNA release pathway [153]. A novel finding of this independent study was that inhibiting or silencing flap-structure-specific endonuclease 1 (FEN1) also suppresses mtDNA release by preventing the production of 500–650 bp oxidized mtDNA linear fragments from circular mtDNA. In addition, this study and others demonstrated that VBIT-4 does not inhibit mPTP opening—an important control—and, instead, promotes mtDNA accumulation in the intermembrane space, as would be expected with block of an MOM transit route [153,154]. Another group found that hexokinase 2 dissociation from the NLRP3 N-terminus, but not mtDNA binding, is required for VDAC oligomerization in macrophages. Supporting a revised role for mtDNA in the signaling pathway [155], their experimental evidence suggests that mtDNA promotes NLRP3 inflammasome activation through recruitment of NLRP3 to mitochondrial VDAC oligomers.

Several additional studies have demonstrated a role for VDAC oligomers in mtDNA release using pharmacological and/or genetic approaches. One study found that the mitophagy E3 ligase Parkin antagonizes VDAC1-mediated mtDNA extrusion via lysine 53 ubiquitination [156]. In a neurodegenerative disease context, another study reported that the ALS-associated protein TDP43 promotes mtDNA release via a potential mPTP-VDAC dependent route, with genetic deletion of downstream STING mitigating disease in an ALS mouse model [41]. In contrast to most studies using only CsA to implicate mPTP opening in mtDNA release, the role of the mPTP was strongly established by complimentary CypD knockout experiments; however, the interpretation of VDAC’s involvement relied on the specificity of VBIT-4’s effects. Finally, in direct contrast to the role proposed for VDAC1/3 oligomers, some evidence suggests that VDAC2 is an important brake on mtDNA release, likely by inhibiting BAK macropore formation [61,157]. Cytosolic mtDNA accumulation and cGAS-STING signaling observed in *Vdac2*-deficient mouse tumor cells were prevented by *Bak1* co-deletion [157]. However, indirect evidence suggests that VDAC2 oligomers may also contribute to mtDNA release in some contexts [155].

## 6. Gasdermin- and MLKL-Mediated Mitochondrial Membrane Pores

Gasdermins represent a superfamily of pore-forming proteins that contribute to the release of mtDNA into the cytosol during both pyroptosis and apoptosis [158,159,160,161,162,163,164], including neuronal pyroptosis after TBI [163]. Initial studies identified gasdermin D (GSDMD) as a mediator of pyroptosis required for interleukin-1 beta (IL-1β) secretion [23,165,166]. It was then shown that an inflammatory-caspase-cleaved GSDMD N-terminal fragment (GSDMD-NT) can form oligomeric pores in membranes [24,25,167,168,169], promote the release of DAMPs like HMGB1 and mtDNA to the extracellular space during pyroptosis [158], and also release cytokines such as interleukin-1 alpha (IL-1α) and IL-1β from living, hyperactivated microglia or macrophages [170,171,172].

Gasdermin D-mediated pore formation is initiated by caspase-mediated cleavage of the autoinhibitory C-terminal domain, which liberates the pore-forming N-terminal domain [173]. The N-terminal fragment inserts into membranes by binding to anionic lipids such as phosphoinositides and cardiolipin [167]. Cleavage of GSDMD is predominantly driven by inflammatory caspase-1, -4, or -5 in humans and caspase-1 or -11 in mice [174].

Platnich et al. in 2018 were among the first to show that pore-forming GSDMD-NT also localizes to mitochondria [175]. They found that the cleaved fragment causes macrophage mitochondrial depolarization and ROS production upstream of NLRP3 activation in response to Shiga toxin 2/LPS co-treatment [175]. Not long after, Rogers et al. reported that GSDMD-NT initiates MOMP and cytochrome *c* release following inflammasome-mediated GSDMD cleavage [176], but the possibility of mtDNA redistribution was not investigated. Subsequently, Huang et al. directly implicated GSDMD-NT in mitochondrial inner membrane depolarization and mtDNA release (Figure 4), showing that intracellular-LPS-induced mitochondrial membrane potential loss and mtDNA release in endothelial cells were abolished by either caspase-11 knockout or GSDMD knockout [159].

De Torre-Minguela et al. further elucidated GSDMD-NT’s roles in pyroptosis in 2021, showing that it is not only required for mtDNA release into the cytosol, but also to the extracellular space [158]. Their study additionally demonstrated that GSDMD-NT overexpression is sufficient to fragment mitochondria and release mtDNA in the absence of NLRP3 inflammasome or caspase-1 activation. Subsequently, Miao et al. demonstrated that PLSCR3, the same cardiolipin-externalizing enzyme important for APOL3 mitochondrial recruitment and permeabilization [81], is also required for GSDMD-NT’s mitochondrial localization and pore-forming activity, with structured illumination microscopy and immunogold electron microscopy providing imaging-based evidence for GSDMD-NT pore formation in both the mitochondrial outer and inner membranes [160].

Other gasdermins, including gasdermin A (GSDMA) [177,178] and gasdermin E (GSDME) [158,176,179], also form mitochondrial membrane pores and release mtDNA in various cell types and contexts. Caspase-3 cleaves GSDME, which can amplify MOMP during apoptosis [158,176], whereas GSDMA may not always require cleavage to form pores [178]. Ras-related protein Rab-5 (RAB5) A triggered the mitochondrial translocation of full-length GSDMA in breast cancer cells, which, upon palmitoylation, formed oligomeric MIM pores in BAX/BAK-permeabilized mitochondria to mediate mtDNA nucleoid release [178]. A Group-A-Streptococcus-secreted protease cleaves GSDMA [180], but whether there are also mammalian proteases that can activate GSDMA pore formation through cleavage is unclear. A 2015 study, one of the first localizing a gasdermin family member to mitochondria, suggested that GSDMA3-N-terminal-fragment-induced cell death requires mPTP opening [181]. However, because a relatively high concentration of CsA (6 µM) was required, BKA was ineffective, and CypD knockout was not investigated, it seems possible that CsA’s protection was through an mPTP-independent mechanism.

Interestingly, a recent study used magnetic-activated cell sorting to demonstrate that at the level of western blot sensitivity, mouse brain GSDMD expression is largely glia-restricted, whereas mouse brain GSDME expression is predominantly neuronal [182]. Through genetic knockout, GSDME was shown to partially mediate TDP-43-induced mitochondrial depolarization [182]. Additionally, GSDME knockout attenuated neurite loss and improved motor neuron survival in ALS models [182]. Although mtDNA release was not investigated in this study, considering these findings in the context of earlier work [41] raises the possibility that the mPTP and GSDME cooperatively contribute to MIM permeabilization in ALS models of disease.

Engineering of the mtDNA-selective fluorescent probe mtNARed recently facilitated stimulated emission depletion super-resolution imaging of mtDNA extrusion in uncoupler or LPS-treated MCF7 cells in real time [183]. Uncoupler concentrations causing complete mitochondrial depolarization triggered mitochondrial swelling, fragmentation, membrane rupture, and the appearance of cytosolic mtDNA within less than 30 min. The mtDNA release was slower during pyroptotic cells death, as mitochondrial swelling accompanied by mtDNA release events were observed after 10 h of LPS treatment. In parallel experiments, GSDMD cleavage was prominent after 24 h of LPS exposure but was not yet detectable by western blot at 12 h post-treatment. A mechanism of swelling-dependent mtDNA release would be consistent with the formation of oligomeric GSDMD-NT mitochondrial inner membrane pores or with sustained mPTP opening. However, whether low-level GSDMD-NT generation, mPTP, both GSDMD-NT and mPTP, or another process was responsible for mtDNA release in either uncoupler-induced release or the 10-h-LPS-treatment-triggered release was not established.

Recent evidence suggests that the pseudokinase mixed lineage kinase domain-like protein (MLKL) can form pores in both the MOM and the MIM during necroptotic cell death [184] (Figure 4). During necroptosis, MLKL is phosphorylated by receptor-interacting serine/threonine-protein kinase 3 (RIPK3) [185,186,187]. Analogous to the gasdermin-mediated mtDNA release mechanism, phosphorylated MLKL (pMLKL) binds cardiolipin in both the MOM and MIM downstream of PLSCR3 phospholipid scramblase activity, forming oligomeric membrane pores enabling mtDNA release into the cytoplasm [184]. Phosphorylated MLKL-mediated MOM and MIM openings measured from images acquired by transmission electron microscopy averaged 300 nm in size, sufficient to allow the passage of mtDNA nucleoids as well as fragments. Supporting that mtDNA was most likely released as nucleoids rather than small fragments, quantitative PCR (qPCR) amplification of several different mtDNA regions from cytosolic mtDNA failed to detect enrichment for any gene sequences [184].

In contrast to the reported MIM herniation through the MOM following BAX/BAK macropore formation, MLKL-induced MOM and MIM breaches appeared similar in size and no MIM herniation was apparent [184]. Intriguingly, although breaches encompassing approximately half the width of individual mitochondria were seen, permeabilized mitochondria generally maintained cristae structure across the non-compromised area. Several disruptors of microtubule structure were identified from a chemical library screen for small-molecule inhibitors of MLKL-induced mtDNA release, including the clinically used compounds colchicine and vincristine [184]. While the investigators hypothesized that MLKL-induced pores could be actively dilated by microtubule-associated motors, how microtubules might facilitate mtDNA release is not yet clear and additional evidence is needed to support the pharmacology-based findings.

It is important to note that the broad-spectrum caspase inhibitor carbobenzoxy-valyl-alanyl-aspartyl-[O-methyl]-fluoromethylketone (z-VAD-fmk) was among the cocktail of compounds added to induce necroptosis in the study demonstrating MLKL-mediated mtDNA release [184]. Caspase inhibition is expected to preclude GSDMD or GSDME mitochondrial pore formation by preventing the production of their porating N-terminal fragments. Whether MLKL also drives mtDNA release to the cytosol in physiological/pathophysiological contexts when an exogenous caspase inhibitor is not present remains to be demonstrated. At present, evidence for direct MLKL-mediated mitochondrial permeabilization remains limited compared with the evidence supporting plasma membrane MLKL function.

## 7. Additional Mechanisms of mtDNA Release

Other than the major gateways listed above, mtDNA release to engage cytosolic DNA sensors can also occur through mechanisms that do not necessarily or exclusively require pores in mitochondrial membranes. Most of these mechanisms involve mtDNA transfer to and release from different membrane-bound intracellular compartments, including autolysosomes, endosomes, and mitochondrial-derived vesicles (MDVs). The mtDNA transfer can occur through the transiting of mitochondria containing DNA or through transport of the mtDNA itself, depending on the pathway.

One incompletely understood mechanism involves dysfunctional mitophagy followed by lysosomal mtDNA escape. To directly test the effect of inhibiting lysosomal DNA degradation on mtDNA signaling, Oka et al. knocked out the acid-dependent lysosomal enzyme deoxyribonuclease (DNase) IIα. DNase IIα deficiency impaired autolysosomal mtDNA degradation and promoted mtDNA-dependent activation of the endolysosomal immune receptor TLR9 in cardiomyocytes [188] (Figure 5a). Whether mtDNA relocalization to TLR9 occurred through a vesicular mtDNA trafficking pathway or involved mtDNA traversing the cytosol is unclear.

In a disease context, systemic lupus erythematosus (SLE) patient monocytes showed impaired autophagic degradation of mtDNA caused by interferon-alpha (IFN-α)-dependent lysosomal deacidification [189]. Although the mtDNA escape pathway was not investigated, in these cells STING knockdown rather than TLR9 knockdown inhibited the downstream mtDNA-dependent immune signaling. STING-dependent signaling was blocked by targeted delivery of an acidity-independent DNAse I—but not by denatured DNAse I—to the lysosomal compartment, supporting a role for defective mitophagy and lysosomal compromise in mtDNA extrusion.

Stimulation with a different cytokine, tumor necrosis factor-alpha (TNF-α), was found to impair mitophagy and increase cytosolic mtDNA in conjunction with cGAS-STING-dependent signaling in the widely used human monocyte cell line THP-1. In the PD-linked PTEN-induced kinase 1 (PINK1)-Parkin canonical mitophagy pathway, PINK1 phosphorylates ubiquitin that is conjugated to MOM proteins, amplifying MOM protein ubiquitination through Parkin E3 ligase activation [190,191,192]. Autophagosomes are then recruited to mitochondria decorated by phospho-ubiquitin to remove these organelles tagged for disposal. A TNF-α-dependent block of mitophagy initiation was demonstrated by showing the near-absence of phospho-ubiquitin with or without addition of a mitophagy inducer and a concomitant depletion of PINK1 from the mitochondrial fraction [193]. While the mechanism of mtDNA leakage to the cytosol was not clarified, cellular respiration was maintained, suggesting that widespread compromise of MIM integrity in the mitochondrial population was unlikely. Further establishing that mitophagy curtails mtDNA-dependent cGAS-STING activation, Jiménez-Loygorri et al. used a model cellular system to demonstrate the ability of the mitophagy inducer urolithin A to prevent cytosolic mtDNA accumulation in a manner prevented by PINK1-Parkin double knockdown [194]. Collectively, these studies, along with others, e.g., [195,196], led to the recognition that mitophagy is a critical checkpoint for inhibiting the initiation of mtDNA-dependent immune signaling while the mechanisms of mtDNA escape require further study.

Aside from the canonical mitophagy pathway, Saunders et al. demonstrated that MIM herniations through BAX/BAK macropores initiate a PINK1-independent form of mitophagy in which MIM proteins are ubiquitinated and the organelle is subsequently delivered to the lysosome [197]. The investigators speculated that this non-canonical mitophagy mechanism may function as an additional checkpoint to prevent mtDNA-dependent inflammatory signaling following incomplete MOMP because its failure could indirectly promote mtDNA release by allowing mitochondria with cytosol-exposed inner membranes to persist in the cell.

MDVs can also participate in mtDNA release into the cytosol (Figure 5b). Trafficking of MDVs to lysosomes for the degradation of damaged mitochondrial components, including mtDNA, is thought to reduce the need for mitophagy and decrease the risk of mtDNA leakage [198]. Todkar et al. identified optic atrophy 1 (OPA1)- and sorting nexin 9 (SNX9)-dependent MDVs as necessary for targeting mitochondrial contents to extracellular vesicles [199]. They further showed that this process can blocked by Parkin, demonstrating that this protein’s role in mitochondrial quality control extends beyond mitophagy [199]. Zecchini et al. reported that elevated levels of fumarate, a metabolite of the tricarboxylic acid cycle, induce the generation of SNX9-dependent MDVs and may facilitate the controlled release of mtDNA and other mitochondrial components [200]. Furthermore, Nguyen et al. demonstrated that GSDME is required for MDV/lysosomal-dependent mtDNA release into the cytosol in human U2OS osteosarcoma cells overexpressing the small ubiquitin-like modifier E3 ligase mitochondrial anchored protein ligase (MAPL), suggesting a secondary role for these pore-forming proteins in mtDNA release beyond creating pores in mitochondrial membranes [201]. In their model system, MDVs deliver cargo to lysosomes. However, rather than mtDNA undergoing degradation, GSDME facilitates mtDNA escape into the cytosol. GSDME cleavage was also detected in bone-marrow-derived macrophages releasing mtDNA following treatment with LPS and interferon-gamma; however, in that case a requirement for GSDME was not formally demonstrated [201]. While the role of MDVs in mediating mtDNA release into the cytosol is actively being defined, further work is needed to clarify how MDVs become compromised, leading to the release of their contents into the cytosol.

In addition to lysosomal mtDNA release, one study suggested that mtDNA can also escape from ruptured endosomes [202]. Newman et al. showed that prolonged mtDNA replication stress during mitochondrial fission results in removal of defective nucleoids through the endosomal pathway [202]. However, upon endosomal rupture, mtDNA is released into the cytosol [202] (Figure 5c). Although the precise mechanism underlying endosomal rupture was not investigated, the authors showed that enlarged mtDNA nucleoids colocalize with both cGAS and galectin 8, a marker of ruptured endosomes, in a RAB5-dependent manner [202]. Lysosomal and endosomal mtDNA release mechanisms, whether by GSDME or other modes of permeabilization, provide yet another potential explanation for how cells can trigger immune signaling without the widespread release of apoptogenic proteins like cytochrome *c* from mitochondria. These explanations are summarized in Table 1.

While Newman et al. showed that mtDNA can escape from ruptured endosomes downstream of prolonged replication stress during mitochondrial fission [202], whether mitochondrial fission and fusion proteins directly participate in the mtDNA release process remains incompletely understood. Multiple studies reported an association between mitochondrial fragmentation and cytosolic accumulation of mtDNA [203,204,205,206,207,208,209]. In the CNS, a decreased level of the mitochondrial pro-fusion protein mitofusin (MFN) 2 in microglia following mouse spinal cord injury correlated with increased cytosolic mtDNA abundance and activation of cGAS-STING signaling [204]. Treatment with the mitofusin-activating small molecule MASM7 [210] attenuated these changes [204], consistent with a potential role for impaired mitochondrial fusion in promoting mtDNA release. However, genetic evidence supporting a direct requirement for mitochondrial fission or fusion proteins in mtDNA release is lacking. One study found that deletion of the mitochondrial fission protein dynamin-related protein 1 (DRP1), the mitochondrial fusion protein OPA1, or both mitofusins (MFN1 and MFN2) failed to prevent mtDNA release during mouse embryonic fibroblast apoptosis induced by inhibition of anti-apoptotic BCL-2 family proteins [79]. Thus, despite recent advances, the extent to which mitochondrial fission and fusion actively regulate mtDNA release in either CNS disease contexts or the periphery remains unclear and warrants further investigation.

Finally, an intriguing mitochondrial RNA (mtRNA)-mediated mtDNA release pathway was recently reported in monocytes that occurred without obvious signs of mitochondrial dysfunction, including depolarization, increased ROS generation, or cytochrome *c* release [211]. Caielli et al. found that both mtDNA and mtRNA escaped from the phagolysosomal compartment of human monocyte-like cells opsonizing red blood cells with retained mtDNA [211], a phenomenon that occurs in SLE [212]. The erythrocyte-derived cytosolic mtRNA then triggered monocyte mtDNA release through an incompletely defined pathway requiring oligomerization of the mitochondrial viral-sensing protein (MAVS) on the MOM [211].

## 8. Targeting Mitochondrial Pores to Limit mtDNA Release

Since mtDNA release is a potential contributor to multiple neurodegenerative diseases with a neuroinflammatory component, there is considerable interest in therapeutically targeting mitochondrial pores to limit its release. BAX activation inhibitor 1, an allosteric BAX inhibitor [213], was shown to reduce mtDNA release in senescent human fibroblasts [57] and, also, to inhibit the release of mtDNA and tunneling nanotube formation triggered by treating human SH-SY5Y or HMC3 immortalized cells with aggregated α-synuclein [214]. Eltrombopag, a Food and Drug Administration (FDA)-approved small molecule that inhibits BAX by competing off BH3-only activator proteins and stabilizing BAX in an inactive conformation [215], impaired senescent cells’ secretion of several cytokines that depend on mtDNA release, including IL-1β [57]. However, eltrombopag’s reported ability to directly activate BAK and induce apoptosis may limit its translatability for neuroinflammatory disease treatment [216]. Additionally, although it can cross the blood–brain barrier, it chelates intracellular iron, which can induce undesirable effects [217]. The newly described covalent BAX inhibitor 3 (CBI-3) reduced spontaneous cell death of induced pluripotent stem cell-derived motor neurons from an ALS patient heterozygous for the TDP-43 G298S mutation but its potential effect on mtDNA redistribution was not investigated [218]. Several other BAX-inhibiting molecules have been identified that inhibit BAX macropore formation at various levels: mitochondrial BAX translocation [219,220,221], BAX channel activity [222,223], and the formation of dimers [224] or oligomers [220,221,225]. However, whether any of these molecules can prevent mtDNA release into the cytosol remains to be determined.

Compounds targeting the mPTP, including CsA, have been shown to reduce mtDNA release into the cytosol in various model systems. Although CsA is FDA-approved, its immunosuppressive actions mediated by calcineurin inhibition rather than mPTP action are undesirable for the neurodegenerative disease target population, most of whom are elderly and vulnerable. The non-immunosuppressive CsA analogues N-methy-valine-4-cyclosporin A (SDZ 220–384/PKF 220–384) [226,227] and N-methyl-4-isoleucine-cyclosporin (NIM811) [228] that inhibit cyclophilin D without inhibiting calcineurin are more attractive drug candidates but translational efforts with these compounds have been unsuccessful to date. More recently, a series of next-generation mPTP inhibitors were identified through screening assays followed by structure–activity relationship optimization [229,230,231,232,233]. A bioRxiv preprint reports that both isoxazole and cinnamic anilide mPTP inhibitors target the recently identified mPTP activator NRLX1 [137], while emerging evidence suggests that triazole derivative compounds may limit mtDNA release by inhibiting Ca^2+^-activated F1Fo ATP synthase [232]. Algieri et al. demonstrated that these triazole derivatives bind to F1Fo ATP synthase and reduce mitochondrial permeabilization via the mPTP without impairing mitochondrial respiration [232]. Although additional studies are needed to assess the efficacy of these compounds in restricting mtDNA release, this work highlights some of the first agents to inhibit potentially core mPTP components other than CypD.

Most studies investigating VDAC1 as a target to block mtDNA release into the cytosol utilized VBIT-4 as a pharmacological inhibitor. However, because VBIT-4 may exert VDAC1-independent effects [152], further work is needed to determine the extent to which VDAC1 itself mediates VBIT-4’s inhibitory effects on mtDNA release. Future studies should also aim to identify additional compounds or targets capable of inhibiting VDAC1 oligomerization and/or pore formation, thereby reducing the likelihood of mtDNA escape through these channels.

Although gasdermins were most recently identified as mitochondrial membrane pore-forming proteins, they are already being investigated as targets to inhibit mtDNA release into the cytosol. Zou et al. demonstrated that treatment with disulfiram, a GSDMD inhibitor, reduced mtDNA release following LPS challenge in human THP-1 monocyte cells [162]. Similarly, Sun et al. showed that dimethyl fumarate, a GSDME inhibitor, prevents GSDME cleavage, a step required for membrane anchoring and pore formation [179]. Additional compounds targeting gasdermins have already been extensively reviewed [234]; future studies should evaluate whether these compounds can not only inhibit gasdermins but also suppress mtDNA release.

Several MLKL inhibitors have been identified and recently reviewed in detail [235], including necrosulfonamide [186] and TC13172 [236]. TC13172, a compound with low nanomolar potency, completely blocks mtDNA release in RIPK3-expressing HeLa cells exposed to a necroptosis-inducing cytokine/drug combination [184].

Finally, cardiolipin represents a promising target for limiting mtDNA release because it plays a central role in multiple pathways that regulate mitochondrial membrane structure and permeabilization. Elamipretide (SS-31; also called Bendavia, MTP-131, or Forzinity) is a mitochondria-targeted tetrapeptide that was recently approved by the FDA for the treatment of Barth syndrome, a multi-system disorder caused by pathogenic mutations in a gene important for cardiolipin synthesis [237,238,239,240]. By binding to cardiolipin, elamipretide prevents cardiolipin oxidation and stabilizes mitochondrial cristae structure [241,242]. In addition to improving mitochondrial bioenergetics and protecting against mitochondrial dysfunction [241], elamipretide has been shown to reduce mtDNA release [243,244], further supporting cardiolipin as an attractive therapeutic target for mitigating mtDNA release.

Elamipretide was reported to suppress mtDNA-cGAS-STING signaling in aged mice, an effect associated with enhanced mitophagy. This finding suggests that cardiolipin-targeted therapies may limit inflammatory mtDNA signaling in addition to improving mitochondrial function [243]. Consistent with a broader effect on mtDNA-dependent inflammatory signaling, elamipretide also reduced activation of the mtDNA-STING pathway in a burn injury model, demonstrating that suppression of mtDNA-dependent inflammation is not unique to one disease model [244]. As one example of elamipretide’s neuroprotective potential, a study found that a 30-min post-treatment with elamipretide attenuated cytochrome *c* release and neuronal cell death while improving sensorimotor deficits in a mouse weight drop model of TBI [245]. Because cardiolipin externalization and remodeling are implicated in multiple mtDNA efflux pathways, including APOL3-, gasdermin-, and MLKL-dependent mechanisms, cardiolipin-targeted therapeutics such as elamipretide could provide a means of simultaneously modulating several otherwise distinct mitochondrial DNA release pathways.

## 9. Conclusions

Since mtDNA release is implicated in numerous neurological diseases and can activate diverse signaling pathways under both homeostatic and pathological conditions, it is imperative to better understand its dynamics. Although several mechanisms have been described, major questions regarding the spatial and temporal dynamics of mtDNA damage, release, and subsequent downstream signaling remain unanswered. Additionally, most foundational mtDNA-release studies were conducted in non-CNS model systems, highlighting a significant gap in the field. Furthermore, because many proposed mtDNA-release mechanisms have been evaluated by measuring mtDNA in cytosolic fractions, methodological limitations should be considered when interpreting results. In particular, many studies quantified mtDNA in cytosolic fractions using qPCR; however, interpretation can be complicated by mitochondrial contamination during fractionation, passive redistribution of mtDNA secondary to cell death, and difficulties distinguishing freely accessible cytosolic mtDNA from mtDNA enclosed within membrane-bound vesicles or extracellular vesicles. Conclusions regarding mtDNA release are stronger when supported by rigorous validation of fraction purity and complementary imaging-based approaches that directly assess mtDNA localization. Table 2 summarizes the model systems, mtDNA-release detection methods, and redistributed mtDNA species reported in several of the key studies supporting the diverse mtDNA-release mechanisms.

Although not all literature is consistent, most evidence suggests that different cell states or death mechanisms are associated with distinct mitochondrial DNA release pathways, e.g., BAX/BAK- and GSDMA- or GSDME-dependent in apoptosis, GSDMD-dependent in pyroptosis, pMLKL-dependent in necroptosis, and mPTP-dependent in senescence. The participation of multiple mitochondrial permeabilization pathways in different cell death modalities is also possible. For example, evidence suggests that GSDMD mediates mitochondrial membrane potential depolarization during necroptotic cell death in mouse bone marrow-derived macrophages harboring the familial Parkinson’s disease G2019S leucine-rich repeat kinase 2 (LRRK2) mutation [246], but MLKL is thought to be required for mtDNA release to the cytosol during necroptosis [184]. Taken together, available evidence suggests that inflammatory mtDNA release is not mediated by a single molecular pore or membrane hole but rather can occur through multiple pore-dependent and pore-independent pathways whose relative importance depends on cell type and pathological context. Notably, PLSCR3-dependent rearrangement of the mitochondria-specific lipid cardiolipin is required for multiple mtDNA efflux pathways, suggesting that targeting upstream cardiolipin alterations could be explored as a method to overcome downstream redundancies. Broadly, therapeutic strategies targeting either mitochondrial pores or these alternative release pathways are attractive approaches for limiting mtDNA release, attenuating inflammatory signaling, and potentially reversing mtDNA release-driven deficits associated with neurological disease.

## Figures and Tables

**Figure 1:**
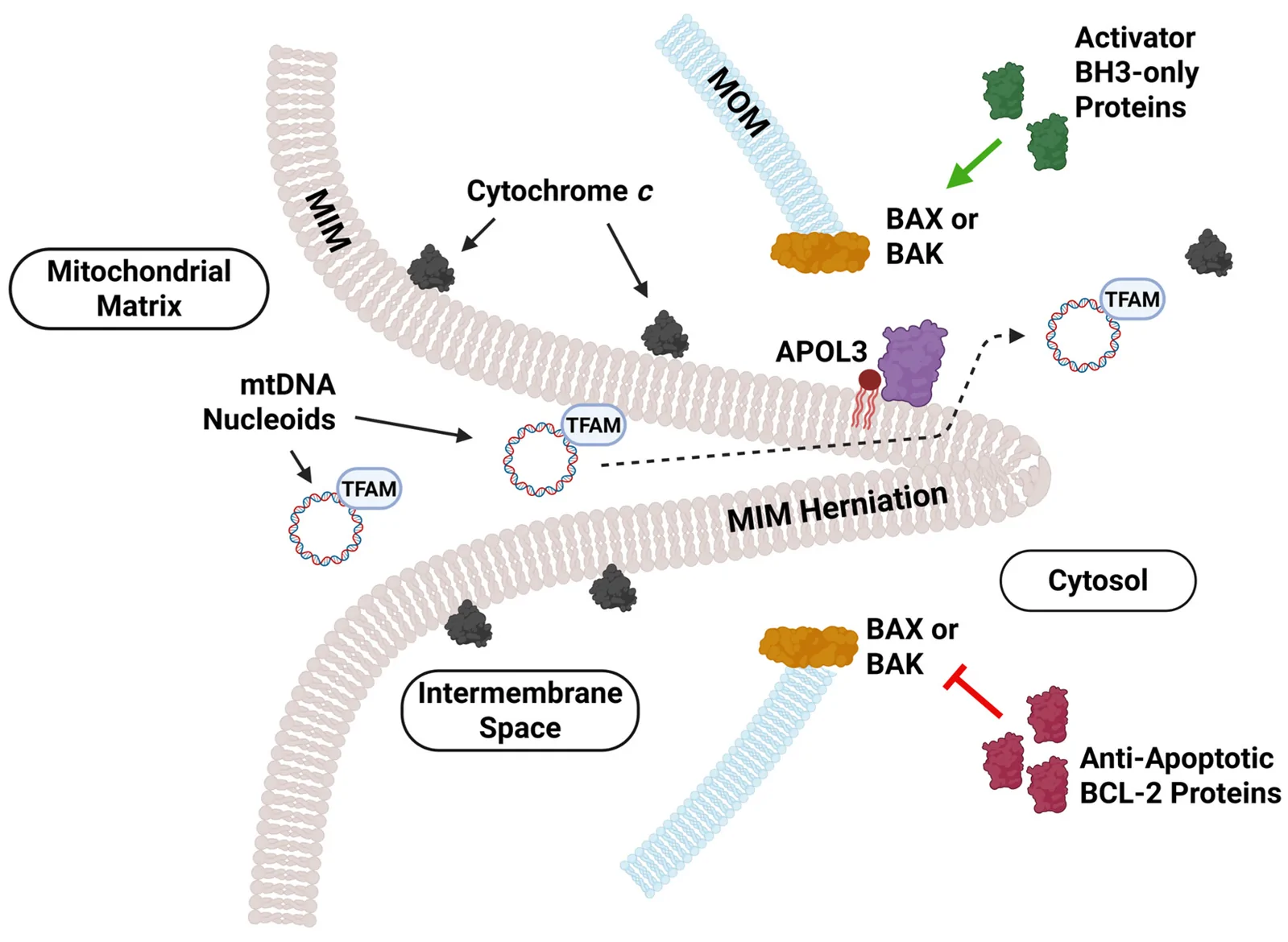
BAX/BAK-mediated macropore formation and mtDNA release. BAX/BAK oligomers form macropores in the mitochondrial outer membrane (MOM), leading to release of cytochrome *c* into the cytosol. Activator BH3-only proteins like BID and BIM promote BAX/BAK oligomerization, whereas anti-apoptotic BCL-2 family proteins inhibit this process. The BAX/BAK macropores also permit the mitochondrial inner membrane (MIM) to herniate through the MOM, a process which facilitates mtDNA escape from some mitochondria by exposing cardiolipin to the cytosol. The cardiolipin-binding protein APOL3 mediates herniated MIM solubilization in lysosome-compromised interferon-gamma primed cells, enabling the release of mitochondrial matrix factors, including TFAM-containing mitochondrial DNA (mtDNA) nucleoids, into the cytosol. The mechanisms resulting in herniated MIM permeabilization during apoptosis are less well defined but appear to be mediated by gasdermin family proteins in some cell types. Created in https://BioRender.com.

**Figure 2:**
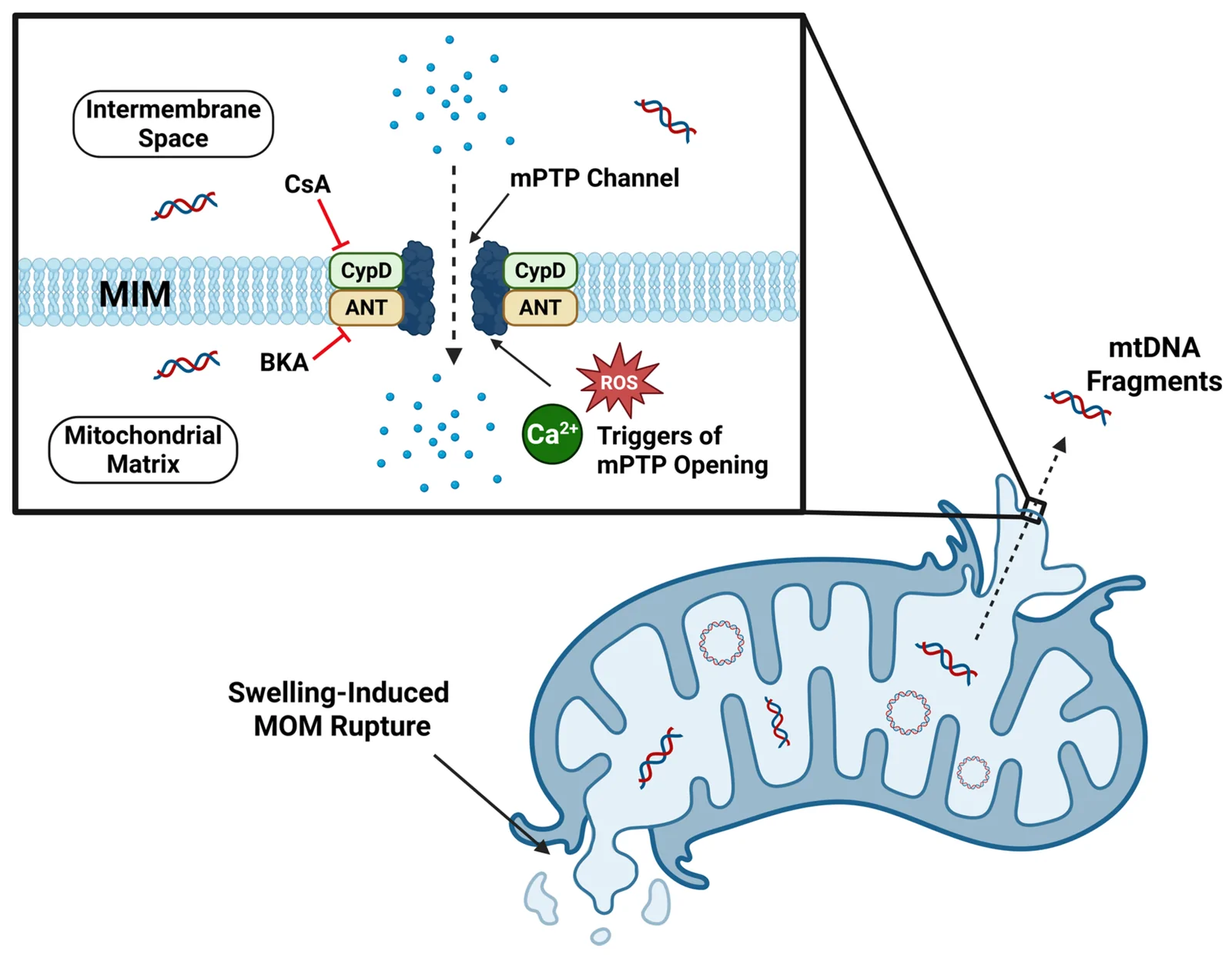
mPTP-mediated MOM rupture and mtDNA release. Elevated mitochondrial matrix calcium and oxidative stress trigger mitochondrial permeability transition pore (mPTP) opening, allowing the escape of ≤700 base pair linear mitochondrial DNA (mtDNA) fragments and many molecules under 1.5 kilodaltons, as well as causing ion and solute entry. During sustained mPTP opening, the level of osmotic water influx into the matrix results in mitochondrial swelling and rupture of the mitochondrial outer membrane (MOM), which enables mtDNA release to pass this second membrane barrier. Some evidence suggests that the mitochondrial inner membrane ruptures after sustained mPTP opening, which may allow the release of mtDNA nucleoids and ≥700 base pair linear mtDNA fragments. Cyclosporin A (CsA) and bongkrekic acid (BKA) desensitize mPTP opening in response to matrix calcium by binding the mPTP regulators CypD and ANT, respectively. The channel-forming mPTP component remains unresolved. Created in https://BioRender.com.

**Figure 3:**
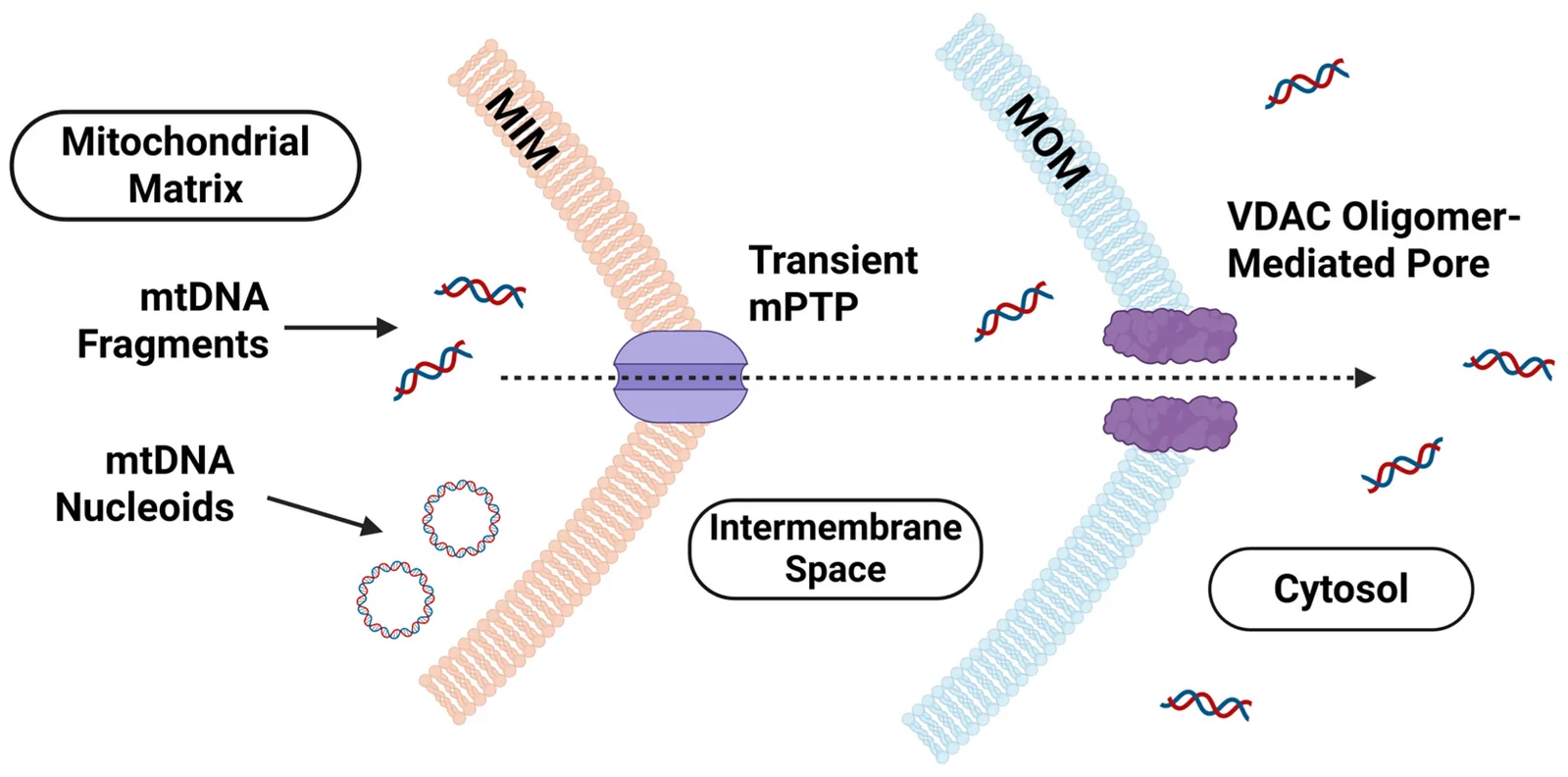
VDAC oligomer-mediated MOM pore formation and mtDNA release. Following stimuli such as oxidative stress and inducers of VDAC-hexokinase 2 dissociation, VDAC monomers oligomerize to form pores in the mitochondrial outer membrane (MOM). Transient mitochondrial permeability transition pore (mPTP) opening increases mitochondrial inner membrane (MIM) permeability to short linear mtDNA fragments of up to about 700 base pairs in size that enter the intermembrane space, which then escape through oligomeric VDAC pores. Created in https://BioRender.com.

**Figure 4:**
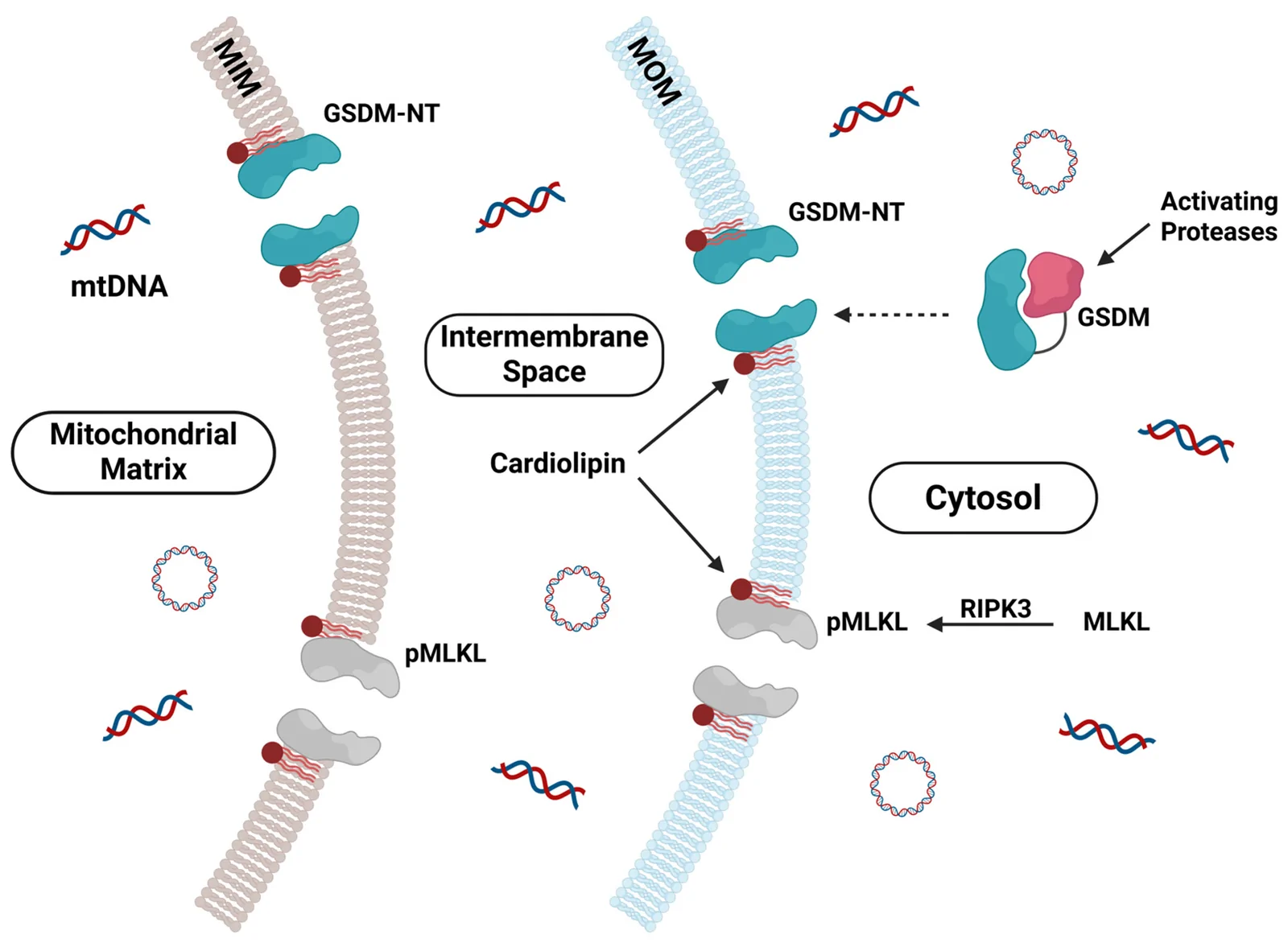
Gasdermin- and MLKL-mediated mitochondrial pore formation and mtDNA release. Gasdermin D (GSDMD) cleavage is mediated by inflammatory caspases 1, 4, 5, and 11, which liberate the N-terminal domain (GSDMD-NT). GSDMD-NT has been reported to insert into both the mitochondrial outer membrane (MOM) and inner membrane (MIM) by binding to cardiolipin, forming oligomeric pores that allow mtDNA to escape into the cytosol. Gasdermin E (GSDME) cleavage to the oligomeric GSDME-NT pore-forming fragment is mediated by apoptotic caspase 3, whereas gasdermin A (GSDMA) may not require cleavage to form pores. Like gasdermins, phosphorylated mixed lineage kinase domain-like protein (pMLKL) binds to cardiolipin in both the MOM and the MIM, forming oligomeric pores that enable the release of mtDNA into the cytosol. Created in https://BioRender.com.

**Figure 5:**
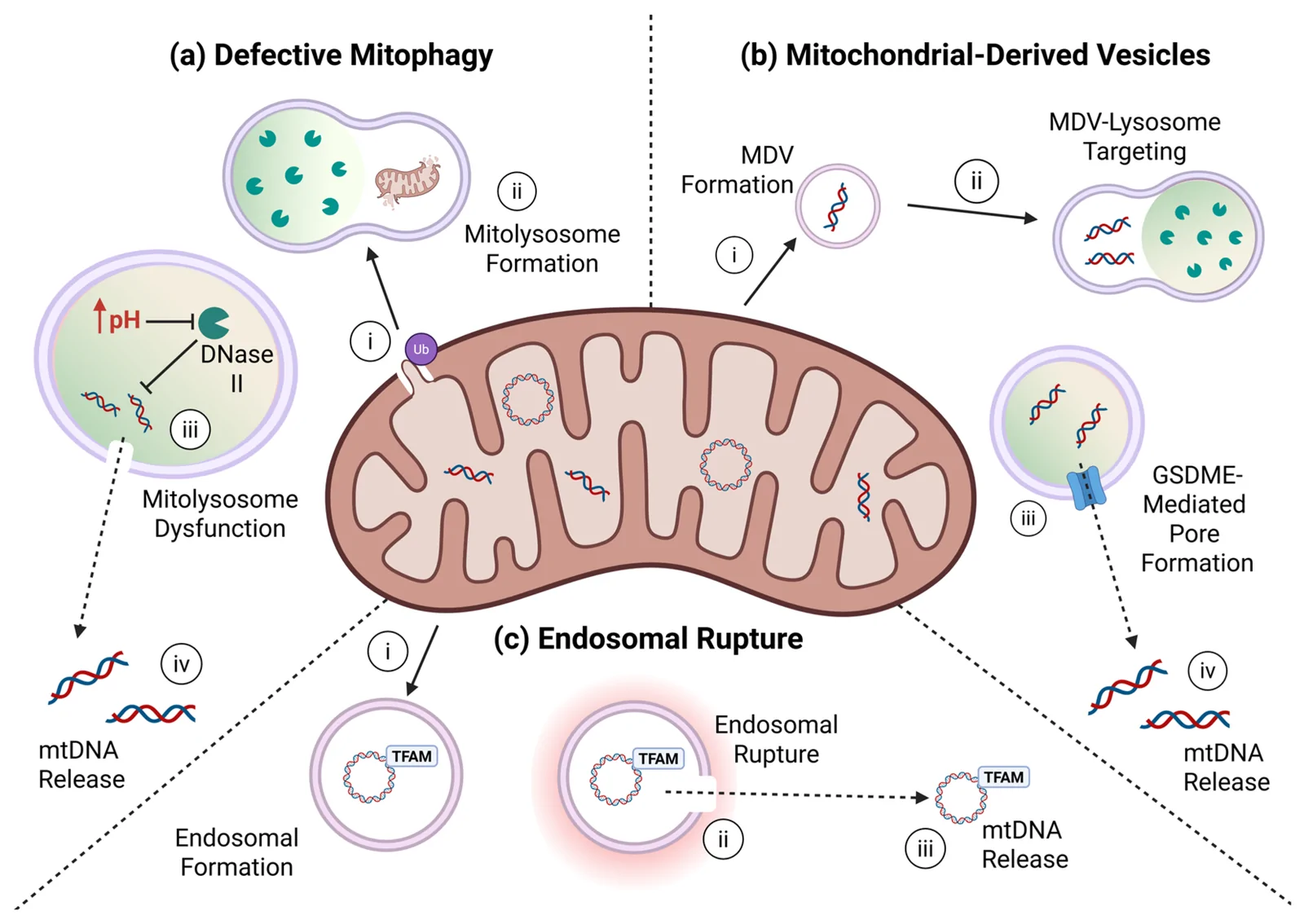
Additional mechanisms of mtDNA release. (**a**) Impaired mitophagy can promote the release of mtDNA into the cytosol. (i) Herniation of the mitochondrial inner membrane (MIM) can trigger MIM ubiquitination, initiating either PINK1-dependent or PINK1-independent mitophagy. (ii) Ubiquitination targets the mitochondrion for lysosomal degradation. (iii) Under normal conditions, the mitochondrial cargo is degraded within the lysosome; however, defects in mitophagosome–lysosome fusion may result in the release of vesicular contents. Alternatively, mitolysosome deacidification can impair DNase II activity, preventing the degradation of mtDNA fragments and facilitating their release. (iv) These processes may enable mtDNA to escape into the cytosol. (**b**) Mitochondrial-derived vesicles (MDVs) can also contribute to cytosolic mtDNA release. (i) Optic atrophy 1 (OPA1)- and sorting nexin 9 (SNX9)-dependent MDVs can contain mitochondrial cargo, including mtDNA. (ii) These MDVs are trafficked to lysosomes. (iii) Upon delivery of mitochondrial contents, GSDME can form pores in the lysosomal membrane. (iv) This process may permit mtDNA escape into the cytosol. (**c**) Endosomal rupture represents another pathway for cytosolic mtDNA release. (i) Chronic activation of a checkpoint that monitors mtDNA replication during mitochondrial fission promotes the trafficking of mtDNA nucleoids into endosomes. (ii) Rupture of endosomal membranes during transport can release endosomal contents. (iii) This process enables mtDNA nucleoids to enter the cytosol. Created in https://BioRender.com.

**Table 1:** Potential explanations for mtDNA-triggered inflammatory signaling without cell death induction.

Proposed Mechanism	Description	References
Cytochrome *c* trapping	Stimuli that activate NLRP3 remodel mitochondrial cristae architecture, appearing to trap cytochrome *c* within cristae compartments to prevent it from triggering cytoplasmic caspase activation.	[58]
Incomplete MOMP	Cytosolic checkpoints such as IAPs prevent caspase activation downstream of moderate cytochrome *c* release.	[82,83]
Transient mPTP-VDAC-mediated mtDNA release	MtDNA fragments traverse transient mPTP and VDAC pores without extensive MOMP or cytochrome *c* release.	[150]
MtDNA escape from lysosomes, endosomes, or MDVs	MtDNA escapes from non-mitochondrial compartment(s) without perturbing the localization of cytochrome *c* or other cell-death-promoting mitochondrial proteins.	[188,201,202]

**Table 2:** Summary of model systems and methods for studies supporting the redistribution of mtDNA through distinct pathways.

Pore	Model Systems	Redistributed mtDNA Species	mtDNA Detection Methods	Reference(s)
BAX/BAK Macropore	Mouse embryonic fibroblasts and human U2OS osteosarcoma cells	TFAM-associated mtDNA nucleoids	TFAM immunofluorescence, TFAM-mClover reporter, DNA immunofluorescence	[79,80]
mPTP	Isolated mouse or rat liver mitochondria and rat kidney mitochondria	mtDNA fragments	PCR amplification and size analysis of released mtDNA fragments	[138,143,144]
VDAC Pore	Mouse embryonic fibroblasts and their purified mitochondria	Short mtDNA fragments	qPCR of cytosolic mtDNA fragments and fragment size analysis	[150]
Gasdermin Pores	Mouse macrophages, endothelial cells, cardiomyocytes, and 4T1 breast cancer cells; human endothelial cells, THP-1 macrophages, HEK293T epithelial cells, and MCF10 mammary epithelial cells	Cytosolic and extracellular mtDNA; nucleoid redistribution reported in select studies	Cytosolic/extracellular mtDNA qPCR, DNA immunofluorescence, nucleoid imaging	[158,159,177,178,179]
MLKL Pore	Mouse L929 fibroblasts and human HeLa epithelial cells	Cytosolic mtDNA in nucleoid-sized structures	qPCR of cytosolic mtDNA genes and size/frequency analysis, DNA immunofluorescence	[184]
MDVs	Human U2OS and 143B osteosarcoma cells and mouse primary macrophages	Unclear	qPCR of cytosolic mtDNA genes, DNA immunofluorescence	[201]
Endosomal Rupture	Human primary lung fibroblast IMR-90 cells and U2OS osteosarcoma cells	TFAM-associated mtDNA nucleoids	Fluorescence in situ hybridization, DNA immunofluorescence	[202]

## Data Availability

No new data were created or analyzed in this study. Data sharing is not applicable to this article.

## Abbreviations

The following abbreviations are used in this manuscript:

AD	Alzheimer’s disease
ALS	Amyotrophic lateral sclerosis
ANT	Adenine nucleotide translocase
APAF-1	Apoptotic peptidase activating factor 1
APOL3	Apolipoprotein L3
ASC	Apoptosis-associated speck-like protein containing a CARD
ATP	Adenosine triphosphate
BAK	BCL-2 homologous antagonist/killer
BAX	BCL-2-associated X protein
BCL-2	B-cell lymphoma 2
BCL-W	B-cell lymphoma W
BCL-X_L_	B-cell lymphoma X long
BH3	BCL-2 homology domain 3
BID	BH3 interacting domain death agonist
BIM	Bcl-2 interacting mediator of cell death
BKA	Bongkrekic acid
bp	Base pair
CBI-3	Covalent BAX inhibitor 3
CNS	Central nervous system
CsA	Cyclosporin A
CypD	Cyclophilin D
cGAS	Cyclic GMP-AMP synthase
DAMP	Damage-associated molecular pattern
DNA	Deoxyribonucleic acid
DNase	Deoxyribonuclease
DRP1	Dynamin-related protein 1
ER	Endoplasmic reticulum
FDA	Food and Drug Administration
FEN1	Flap-structure-specific endonuclease 1
FTD	Frontotemporal dementia
GSDMA	Gasdermin A
GSDMD	Gasdermin D
GSDMD-NT	Gasdermin D N-terminal fragment
GSDME	Gasdermin E
HMGB1	High mobility group box 1
IAP	Inhibitor of apoptosis protein
IL-1α	Interleukin-1 alpha
IL-1β	Interleukin-1 beta
IFN-α	Interferon-alpha
LPS	Lipopolysaccharide
LRRK2	Leucine-rich repeat kinase 2
m-3M3FBS	2,4,6-trimethyl-N-(m-3-trifluoromethylphenyl) benzenesulfonamide
MAPL	Mitochondrial anchored protein ligase
MASM7	Mitofusin activator small molecule 7
MAVS	Mitochondrial anti-viral signaling protein
MCL-1	Myeloid cell leukemia 1
MDV	Mitochondrial-derived vesicle
MFN	Mitofusin
MIM	Mitochondrial inner membrane
MLKL	Mixed lineage kinase domain-like pseudokinase
MOM	Mitochondrial outer membrane
MOMP	Mitochondrial outer membrane permeabilization
mPTP	Mitochondrial permeability transition pore
mtDNA	Mitochondrial DNA
mtRNA	Mitochondrial RNA
NEK7	NIMA-related kinase 7
NINJ1	Ninjurin-1
NLRP3	NLR family pyrin domain containing 3
NLRX1	NLR family member X1
NR4A1	Nuclear receptor subfamily 4 group A member 1
NUR77	Neuron-derived clone 77
OPA1	Optic atrophy 1
PAMP	Pathogen-associated molecular pattern
PCR	Polymerase chain reaction
PD	Parkinson’s disease
PiC	Mitochondrial phosphate carrier
PINK1	PTEN-induced kinase 1
PLSCR3	Phospholipid scramblase 3
pMLKL	Phosphorylated mixed lineage kinase domain-like pseudokinase
qPCR	Quantitative PCR
RAB5	Ras-related protein in brain 5
RIPK3	Receptor-interacting serine/threonine-protein kinase 3
RNA	Ribonucleic acid
ROS	Reactive oxygen species
SLE	Systemic lupus erythematosus
SNX9	Sorting nexin 9
STING	Stimulator of interferon genes
TBI	Traumatic brain injury
TDP-43	TAR DNA-binding protein 43
TNF-α	Tumor necrosis factor-alpha
TFAM	Transcription factor A, mitochondrial
TLR	Toll-like receptor
VDAC	Voltage-dependent anion channel
z-VAD-fmk	carbobenzoxy-valyl-alanyl-aspartyl-[O-methyl]-fluoromethylketone
